# The Efficacy of a Diode Laser on Titanium Implants for the Reduction of Microorganisms That Cause Periimplantitis

**DOI:** 10.3390/ma14237215

**Published:** 2021-11-26

**Authors:** Anna Wawrzyk, Michał Łobacz, Agnieszka Adamczuk, Weronika Sofińska-Chmiel, Mansur Rahnama

**Affiliations:** 1 Silesian Park of Medical Technology Kardio-Med Silesia in Zabrze, M. Curie Skłodowskiej 10C, 41-800 Zabrze, Poland; 2 The Chair and Department of Oral Surgery, Medical University of Lublin, Chodźki 6, 20-093 Lublin, Poland; michallobacz@tlen.pl (M.Ł.); rahnama.m@interia.pl (M.R.); 3 Institute of Agrophysics, Polish Academy of Sciences, Doświadczalna 4 Str., 20-290 Lublin, Poland; agn.adamczuk@gmail.com; 4 Analytical Laboratory, Institute of Chemical Sciences, Faculty of Chemistry, Maria Curie Skłodowska University, Maria Curie Skłodowska Sq. 2, 20-031 Lublin, Poland; weronika.sofinska-chmiel@mail.umcs.pl

**Keywords:** titan implant, diode laser, decontamination, microorganisms, periimplantitis

## Abstract

The paper presents the optimisation of a safe diode laser irradiation process applied to the surface of titanium implants in order to reduce microbial numbers in the treatment of inflammation classified as periimplantitis. The study comprised isolation and identification of microorganisms inhabiting surfaces of dental implants, crowns, teeth and saliva from patients with fully symptomatic periimplantitis. Microorganisms were detected by a culture-dependent method and identified with the use of MALDI-TOF mass spectrometry. The isolated microorganisms were inoculated on the surface of a new implant and then irradiated by a diode laser (wavelength of 810 ± 10 nm) in one, two or three repetitions and biocidal efficacy was assessed. To evaluate impact of laser irradiation on roughness, morphology and structure of the implant surface, optical profilometry, scanning electron microscopy and optical microscopy were used. Examination of the tested surfaces and saliva revealed the presence of Gram-positive and Gram-negative bacteria and one fungal species. In all patients, cultures from the endosseous part of the implant revealed the presence of the pathogenic and pyogenic bacterium *Streptococcus constellatus*. In 13 out of 20 samples laser-irradiated in duplicate and triplicate, all microorganisms were eliminated. The irradiation used did not cause any changes in the properties of the implant surface.

## 1. Introduction

Periimplantitis is a pathology of the tissues around a dental implant that may be related to the presence of microorganisms. It is characterised by progressive bone loss accompanied by inflammation of the connective tissue around the implant. The disease process in periimplantitis can occur even at an early stage of implant treatment, and so far evidence suggests that the course of the disease varies depending on patient predisposition. It is rare for clinically asymptomatic disease to involve progressive bone loss around the implant [1]. Al-Sabbagh et al. [2] characterised and classified the condition of periimplant tissues as the following: periimplant health, periimplant mucositis, periimplantitis, and periimplant soft tissue and bone tissue deficiencies leading to the loss of structural and functional connection with the bone. The risk of developing periimplantitis is increased in patients who have a history of chronic periodontitis, poor plaque control, and no regular maintenance care after the implant therapy is completed [3]. The earlier the diagnosis and treatment initiation, the better the chance of success. 

Dental implant therapy has become a routine procedure in dental surgery. Nevertheless, the numbers of patients affected with periimplant inflammation is consistently on the rise. Currently, there are no established, uniform procedures to prevent peri-implant inflammation; it is believed, however, that prophylaxis and proper oral hygiene are the most important measures. Jepsen et al. [4] estimated the weighted mean prevalence for periimplant mucositis as 43% (CI: 32–54%), while for periimplantitis the figure was 22% (CI: 14–30%). The same study also revealed that patient-administered mechanical plaque control with toothbrushes can be considered an effective preventive measure. The authors also emphasized the need for surgeons to ensure that the implant position is adequately designed and the prosthetic suprastructure is shaped in a way that allows for proper personal cleaning and professional plaque removal. A number of researchers, e.g., Serino et al. [5], underline the importance for dental professionals to educate their patients who are receiving dental implants about proper oral hygiene. 

Periimplant and periodontal microbiomes are microbiologically distinct ecosystems [6]. Cosyn et al. [7] demonstrated that in some cases, even without clinical symptoms of periimplantitis, the surfaces around the implant house a strikingly high number of pathogenic microbial species, and the most considerable contamination is most probably present in the crevices along the implant-abutment interface and the abutment-prosthesis interface. Contamination with microorganisms is also present on the surfaces around implants, e.g., on the healing abutments used in the first stage of implant treatment [8]. The most effective way to prevent periimplantitis is through preventing infection by ensuring daily oral hygiene and keeping the area around the implant clean.

In treating periimplantitis, clinicians currently opt for mechanical debridement and surgical treatment, but there is no one verified treatment procedure that would be considered the best choice in this indication. Still, advanced methods of surface decontamination are continuously sought after to eliminate microbial growth. One of such methods is diode laser irradiation in an optimised process.

Lasers are increasingly finding their way into dentistry and dental surgery as a method of decontamination and, alternatively, the treatment of soft and hard tissues, as they can deliver either very low or very high concentrated power to a specified point [9]. Attempts were made to test the biocidal effectiveness of lasers, but the irradiation doses and species of microorganisms differed from those included in the present study.

Porcel et al. [10] tested the biocidal efficacy of Er: YAG against *Enterococcus faecalis* in root canal. Kasic et al. [11] tested the Nd YAG laser against *E. faecalis* and *Candida albicans* in root canal, a Maden et al. [12] checked the effectiveness of the Nd: YAG laser on 7 strains of fungi in dentina. According to Chala et al. [13], who conducted a review of laser types used so far in clinical practice, not every type of laser can be used for surface decontamination. Nd:YAG lasers are not recommended for implant decontamination since they pose a risk of partial melting and cracking of surfaces irrespective of the initial power levels [14]. Research confirms that titanium implants are not damaged by diode lasers and that these lasers can be effectively used for decontaminating their rough surfaces. There is still the risk that excessive heat can be produced by this type of laser in the bone around the implant, but only if incorrect, poorly optimised irradiation parameters are applied and the procedure is suboptimally performed [15,16]. Any interference can contribute to an increase in roughness and create good conditions for the formation of a biofilm.

The aim of the research was to adjust the diode laser to irradiate the dental implant in a way that eliminates microorganisms and at the same time does not increase the roughness of the titanium surface.

## 2. Materials and Methods

### 2.1. Patients and Materials

The study described 3 male patients aged 65 (patient 1), 69 (patient 2), and 82 (patient 3), who were non-smokers, had no systemic conditions and did not take any medication, but had poor oral hygiene habits. The evaluation of patients participating in the study was based on the measured aproximal plaque index (API), pocket probing depth (PPD) and bleeding on probing (BoP). The API of the patients under study was between 75–100%, which indicates poor oral hygiene. The PPD of the examined patients was >4 mm, the measurements in the area of the examined implants ranged between 6 and 9 mm. BoP in the area of the examined implants was positive (+).

Each patient presented with similar symptoms of advanced periimplantitis, namely the following: pain on palpation of the area, soft tissue redness, unpleasant odour, purulent exudate from the gingival crevice and radiologically confirmed bone loss around the abutment of the implant. The implants under examination had been placed in positions: 13 for 22 years (patient 1), 44 for 8 years (patient 2), and 36 for 7 years (patient 3). The implants were completely removed and examined. The implants affected with periimplantitis were all made from the Ti-6Al-4V extra low interstitial titanium alloy (HS7-3). 

In order to assess the environment of the implants under examination, a 1 mL sample of saliva was collected from each of the patients, and a swab was obtained with a brush from the surface of the dental crown installed over the implant. Subsequent swabs were collected from the abutment before surgical exposure and from the endosseous fixture of the explanted implant. The cultured microorganisms isolated from the swabs, multiplied and identified to the species level, were frozen and cryobanked at a temperature of −80 °C. Then, they were revived and used to inoculate new implants.

In order to test the biocidal efficacy of the laser, sterile titanium implants made of Ti-6Al-4V ELI (Alpha-Bio) were inoculated with the microorganisms at the level of abutment and endosseous fixture and subsequently irradiated with a laser in three variants. The selected test surfaces of the implants were the ones that are most often exposed in cases of periimplant inflammation and periimplantitis.

### 2.2. Microbiological Methods

#### 2.2.1. Assessment of Microbiological Contamination

Swabs were taken from the surface of the crowns and from the components of the implants, which were afterwards each placed in 10 mL of sterile saline solution and shaken for an hour, and a 10-fold dilution series were made. Using a 0.5 mL automatic pipette, 0.5 mL of saliva was collected from each patient and mixed with 9.5 mL of saline solution. For the purpose of microbial culturing, 0.3 mL of initial suspension and serial decimal dilutions was inoculated on 3 plates of enriched agar medium containing 5% sheep blood (Columbia Blood Agar, Oxoid) and incubated in aerobic conditions with 5% CO_2_ for 48 h at a temperature of 36 ± 2 °C. The same amounts of initial suspension as for bacteria were used in testing the presence of fungi and inoculated on the Sabouraud agar (Oxoid) medium, and then incubated for 5 days at a temperature of 25 ± 2 °C. The presence of anaerobic bacteria was detected by inoculation on Scheadler anaerobe agar with horse blood (oxoid) and incubation in anaerobic conditions at 36 ± 2 °C for 4 days. Next, every colony that grew on the media was isolated and identified using matrix-assisted laser desorption/ionisation time-of-flight mass spectrometry (MALDI-TOF MS, (Bruker Daltonics, Germany).

#### 2.2.2. Laser Irradiation

The study used a diode laser (Elexxion AG, Singen, Germany) with a wavelength of 810 nm ± 10 nm. The variant used (L) was at a pulse-output of 25 W, a frequency of 15,000 Hz, a pulse-duration of 10 µs, a mean-output of 3.84 W, and a power-density of 9.60 W/cm^2^. The optical fibre diameter used was 600 µm. The implant surfaces were irradiated in 3 variants: L1: 1 × 15 s, L2: 2 × 15 s, L3: 3 × 15 s at a distance of 0.5 mm from samples. Between irradiation sessions, an interval of 1 min was used.

#### 2.2.3. Assessment of Decontamination Effectiveness

In order to optimise the biocidal efficacy of laser irradiation, surfaces of sterile titanium implants were inoculated with a single species of microorganisms isolated from the tested implant surfaces, crowns and saliva, which had been cultured on Columbia agar. Then freshly grown bacteria harvested from plate inocula were prepared with sterile loop. The microorganisms were placed in sterile saline solution (0.9% NaCl) and inocula were optimized to the concentration present on the surface of examined implants with the application of densitometric or plate method. A total of 20 µL was inoculated on the endosseous fixture and abutment in 5 aliquots of 4 µL, each time allowing it to dry. The tests were repeated three times. Control samples were not exposed. The number of microorganisms before and after laser irradiation was defined with the same method which was applied for the evaluation of the implants’ contamination.

The efficacy of laser irradiation was tested on the following selected microorganisms: 4 strains of Gram-positive bacteria, 4 strains of Gram-negative bacteria and 2 species of fungi, which were considered representative for their respective microbial groups. In addition, to monitor the quality of the study, laser efficacy was also tested against representative microorganisms from each group obtained from the recognised American Type Culture Collection (ATTC). For Gram-positive bacteria, the strain used was *Staphylococcus aureus* ATCC 29213; for Gram-negative bacteria, the selected representative was *Escherichia coli* ATTC 25922; and the representative species of fungi was *Candida albicans* ATTC 10231.

#### 2.2.4. Statistical Analysis

The collected data were statistically compiled. Averages and standard deviations were calculated. The assessment of statistical significance of the differences regarding reductions in microbial numbers was made with ANOVA (one-way analysis of variance) and the least significant difference (LSD) test. The Statistica 6.0 software (Statsoft, Tulsa, OK, USA) was used for calculations (a significance level of *p* < 0.05).

### 2.3. Methods of Surface Analysis

In order to assess the impact of laser irradiation on the implant surface, analysis was performed with the use of optical microscopy, scanning electron microscopy (SEM) and optical profilometry. The tests were carried out for a randomly selected sample before the laser irradiation process, control samples and after laser irradiation in 3 variants L1, L2 and L3.

#### 2.3.1. Optical Microscopy

The surface of each prepared sample was examined microscopically at multiple points before and after laser irradiation. The surface imaging of the tested materials was performed using the Eclipse MA 200 (Nikon, Tokyo, Japan) optical microscope with the D-Eclipse C1 confocal attachment. In order to determine the nature of changes in the chemical structure as well as the topography and surface morphology, photomicrographs of the examined objects in reflected polarized light and fluorescence images of the same objects were made using a confocal attachment with a violet laser with a wavelength of λ = 405 nm. 

#### 2.3.2. Scanning Electron Microscopy

A scanning electron microscope FEI Quanta 3D FEG (Thermo Scientific, Waltham, MA, USA) was employed to examine the implant surface structure before and after laser irradiation and to evaluate an impact of laser irradiation on the surface morphology of the implant. The implants were deposited onto aluminium specimen mounts with self-adhesive carbon conducting discs and coated with an Au/Pd alloy by using Polaron SC7640 sputter coater (Quorum Technologies, Lewes, UK). The microscopic images were obtained at a high vacuum (HV) with topographic contrast, i.e., the registration of secondary electrons (SE) escaping the very thin surface layer of the specimen. The micrographs of the surface of the study specimens were registered at a magnification range of 100 to 5000×. This paper presents comparative results for sample SEM images registered at magnifications of 500 and 5000×.

#### 2.3.3. Optical Profilometry

In order to assess the influence of the laser on the surface of the tested material, analyses were performed using optical profilometry. Roughness measurements of the tested materials were made using the Contour GT-K1 optical profilometer (Veeco, Plainview, NY, USA). The tests were carried out using the VSI (vertical scanning interferometry) technique. The maximum scanning height in this technique is 10 mm.

Linear profiles in the X axis and the Y axis were also made and the following roughness parameters were determined: the arithmetic mean of the elevation profile Ra and the mean square elevation profile Rq and the maximum height of the profile Rt. The roughness parameters were determined for three areas of the test samples for the following scan sizes: 117.2 µm × 156.3 µm and 45 µm × 62 µm. This paper presents the results of the research performed for a larger scan area: 117.2 µm × 156.3 µm. In order to determine the roughness parameters correctly, the sample inclination was corrected, and the waviness of the surface was taken into account.

The roughness measurement (Ra) uncertainty was estimated while taking into account the repeatability, recovery, de-calibration of the apparatus and pattern uncertainty. The uncertainty was estimated for the two extreme points of the Ra measurement range (upper and lower limits) with the assumption of a trapezoidal distribution.

## 3. Results

### 3.1. Assessment of Microbiological Contamination and Identification of Microbial Species from the Implants, Crowns and Saliva

Among aerobic microorganisms detected on the study surfaces (Table 1), we identified the following Gram-positive bacteria: *Staphylococcus aureus*, *Streptococcus constellatus*, *Streptococcus oralis*, *Streptococcus pneumoniae*, *Rothia mucilaginosa*, and *Rothia aeria*, and the following Gram-negative bacteria: *Haemophilus parainfluenzae*, *Klebsiella pneumoniae*, *Klebsiella oxytoca*, and *Veilonella parvula*. One fungal species—*Candida guilliermondii*—was also cultured from the study samples, as well as one anaerobic species of bacteria—*Actinomyces odontolyticus*.

The most numerous members of the microflora inhabiting the surface of the implants and the surrounding environment were bacteria from the family Streptococcaceae and the genus *Rothia*. The highest species diversity was found to exist on the abutment and in the saliva in patient 2 (7 and 7 species, respectively). The lowest species diversity was identified on the endosseous fixture in patient 3 (1 species). Totaling all the microorganisms detected in the samples collected from all three patients, the lowest species diversity was found on the endosseous fixtures, and the highest number of species was identified on the surface of the crowns constituting the suprastructures of the implants. 

In all the samples apart from the endosseous fixture of patient 3 we identified an opportunistic bacterium *H. parainfluenzae* and pathogenic Streptococci. The pathogenic and pyogenic bacterium *Streptococcus constellatus* was cultured from the samples of all three patients and was also detected in their saliva. In the case of patient 3, this pathogenic and opportunistic microorganism completely dominated all the other species.

Most microbial species isolated in this study are part of natural oral microflora according to the Human Oral Microbiome database [17].

However, there were also microorganisms that normally inhabit e.g., the nasal cavity, such as *H. parainfluenzae*, *S. pneumoniae*, and *S. aureus*. *E. faecalis* and *K. oxytoca*, also identified among the isolated species, are usually found in the gastrointestinal tract, where they can cause diseases, while *K. pneumoniae* is a pathogen of the respiratory tract; they all exhibit antibiotic resistance.

### 3.2. The Reduction of Microorganisms on the Implant after Using Three Dose Variants of Diode Laser

All differences between the unirradiated samples and irradiated with laser in different variants were statistically significant (* in Table 2).

Percent reduction in the number of microorganisms on the abutments and the endosseous fixtures in all irradiation variants was 86–100% for bacteria and 71–100% for fungi. The more irradiations of 15 s each were applied, the proportionately greater was the percent reduction in all microorganisms for both irradiated parts of the implant. A 100% reduction was achieved in 13 out of 20 samples irradiated with variant L3, in 10 samples irradiated with variant L2, and in 8 samples irradiated with variant L1. Laser L3 eliminated completely all the species of Gram-negative bacteria and 4 out of 8 tested species of Gram-positive bacteria; in 1 of 4 samples, the laser also eliminated the fungus. Gram-negative bacteria were eliminated at a rate of 86% (*K. oxytoca*) on the abutment when irradiated with variant L1 of the laser for 15 s) to 100% (all microorganisms irradiated with variant L3, both on the abutment and the endosseous fixture).

*E. coli* obtained from the widely recognised strain collections of ATCC was eliminated in 100% of all tested irradiation variants except L1 on the endosseous fixture (99.99%). As regards Gram-positive bacteria, the lowest reduction percentage was achieved for *S. pneumoniae* when irradiated on the endosseous fixture. None of the laser doses managed to completely eliminate *R. mucilaginosa* on either part of the implant and *S. pneumoniae* and *S. constellatus* on the endosseous fixture.

Neither did any of the laser doses applied on either part of the implant eliminate 100% of the fungi isolated from the patients. Resistance to laser irradiation was shown by the fungus *C. guilliermondii*, which was isolated from the abutment of patient 2 (71.01%). Another fungus, *Candida albicans* ATTC, showed 100% susceptibility even with L1.

*S. aureus* ATTC was eliminated in 100% already after 15 s of irradiation on both parts of the implant. All the tested microorganisms from the recognised microbial culture collection ATCC were eliminated in 100% with each dose of the laser when irradiated on the abutment. The lowest reduction of 99.99% with L1 on the endosseous fixture was achieved for *E. coli*.

For 7 out of 10 tested microorganisms, one dose of the laser was 100% effective on the abutment. In each case, L2 and L3 laser irradiation proved equally or more effective on the abutment than on the endosseous part of the implant.

### 3.3. Morphological Analysis of the Surface of the Implants

#### 3.3.1. Confocal Microscopy

**Figure 1:**
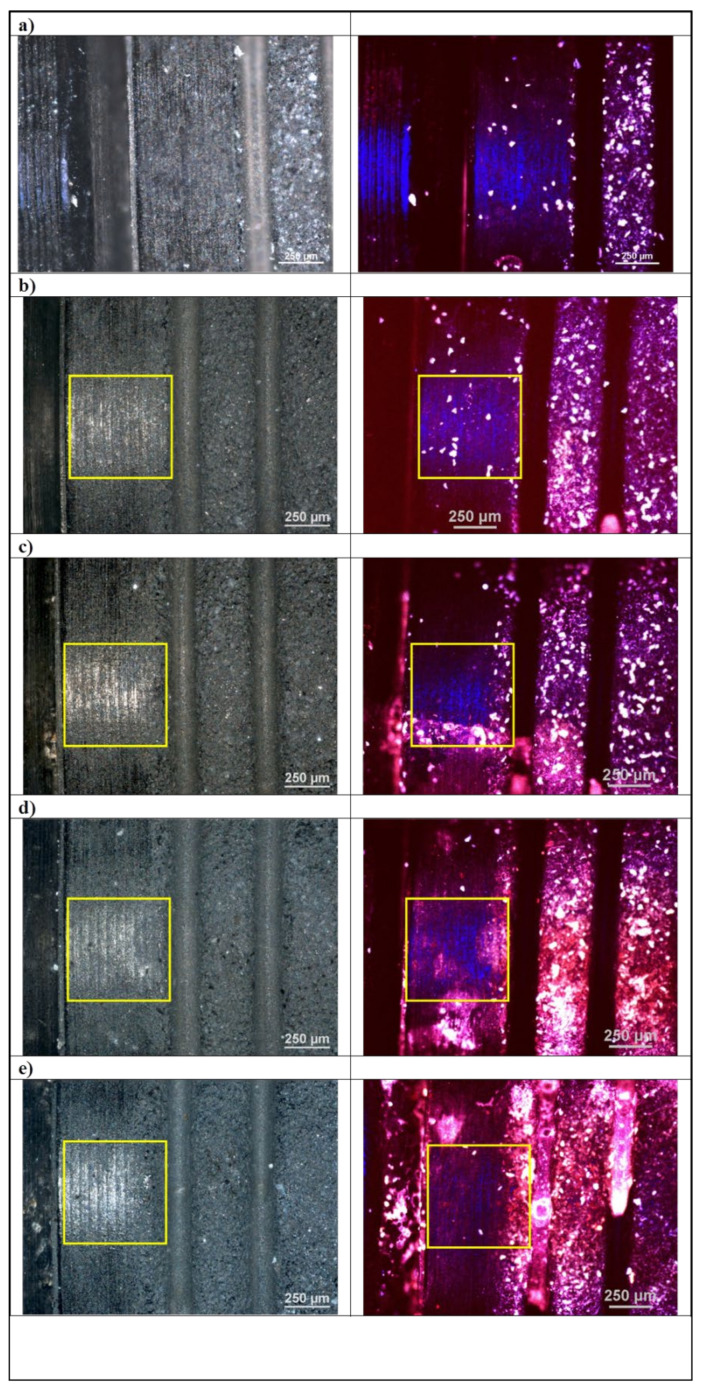
Optical and confocal microscopy. Micrograph of the implant sample surface (**left column**) under optical microscope and (**right column**) under confocal microscope, laser wavelength λ = 405 nm. (**a**) randomly selected site, (**b**) control, (**c**–**e**) disinfection variants. Laser application sites are marked with a frame.

In order to assess the surface of the tested material before and after the laser irradiation process, microscopic examinations were performed using confocal microscopy. The photo shows microscopic photos taken in reflected polarized light (left column) and with the use of a laser with a wavelength of λ = 405 nm (right column). The test results are shown in Figure 1.

The conducted microscopic examinations with the use of optical microscopy showed changes in fluorescence under the influence of laser light. Studies have shown that with each higher variant of the laser, the absorbance of the radiation increases, and the fluorescent light shifts towards the red. It should be assumed that there is a laser cleaning effect on the surface of the tested dental implants. This is confirmed by microbiological tests and SEM images. Optical microscopy studies did not show any destructive effect of laser radiation on the surface of dental implants.

#### 3.3.2. Scanning Electron Microscopy

**Figure 2:**
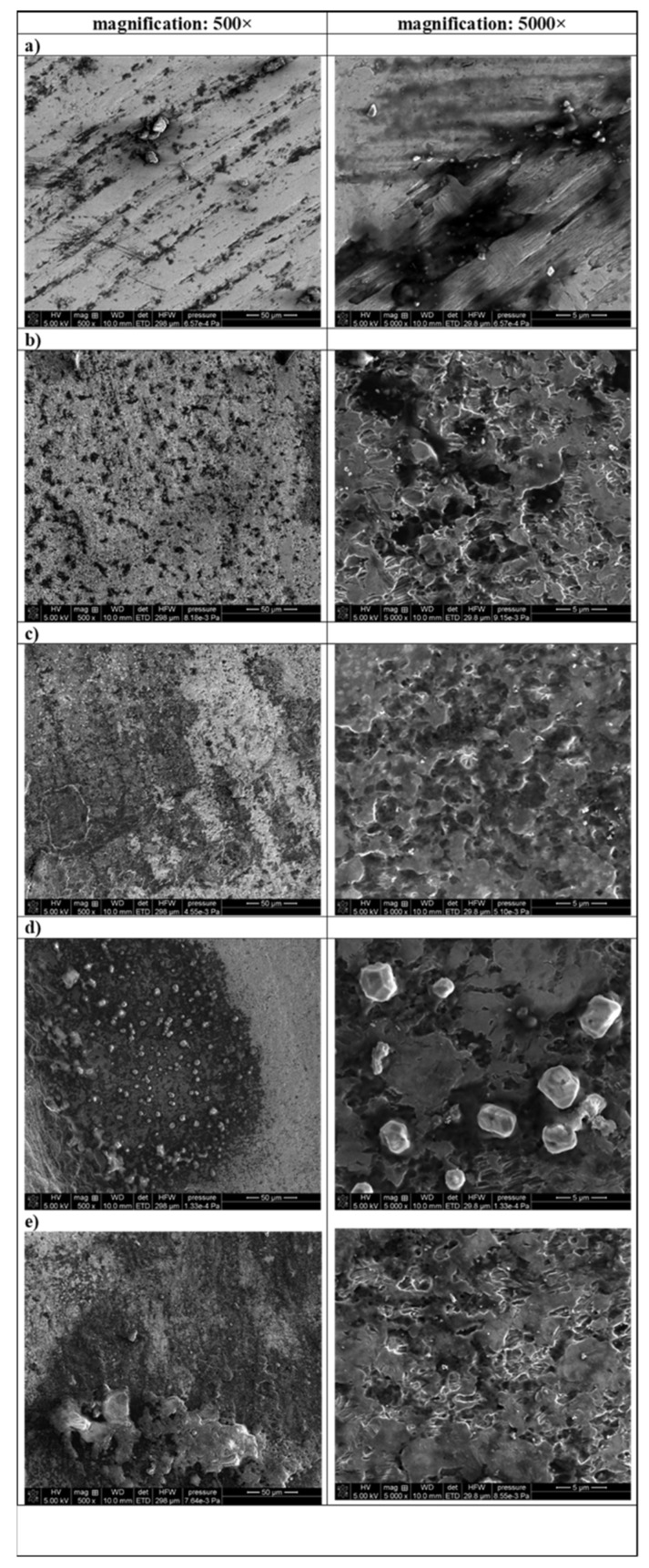
SEM images of the surfaces of titanium implant samples: (**a**) randomly selected site, (**b**) control, (**c**) L1: 1 × 15 s, (**d**) L2: 2 × 15 s, (**e**) L3: 3 × 15 s.

In order to evaluate changes in surface morphology, SEM analysis was performed. A set of SEM images showing the topography and morphology is presented in Figure 2.

It can be seen from Figure 2 that the surface of the titanium implant samples, both before laser irradiation and after, have a fractal, rough structure. A lot of irregularities such as cracks, grooves (randomly selected site) and irregular hollows (in the case control samples and samples after disinfection) can be observed. The sample of randomly selected sites were characterized of smoothest surface.

The SEM images clearly show the cleaning effect of the laser. Comparing images made at magnification 500× it can be observed that the more irradiation sessions, the less contamination is visible on the surface. On the surface of the sample after decontamination with L3 laser, the number of black spots is at its lowest. The exception is a sample treated with L2 laser dose, where great irregular white particles appear. 

Based on the results of SEM analysis it can be stated that non-destructive effects of laser treatment (with all 3 variants) were noticed on the surface of implants.

#### 3.3.3. Optical Profilometry

**Figure 3:**
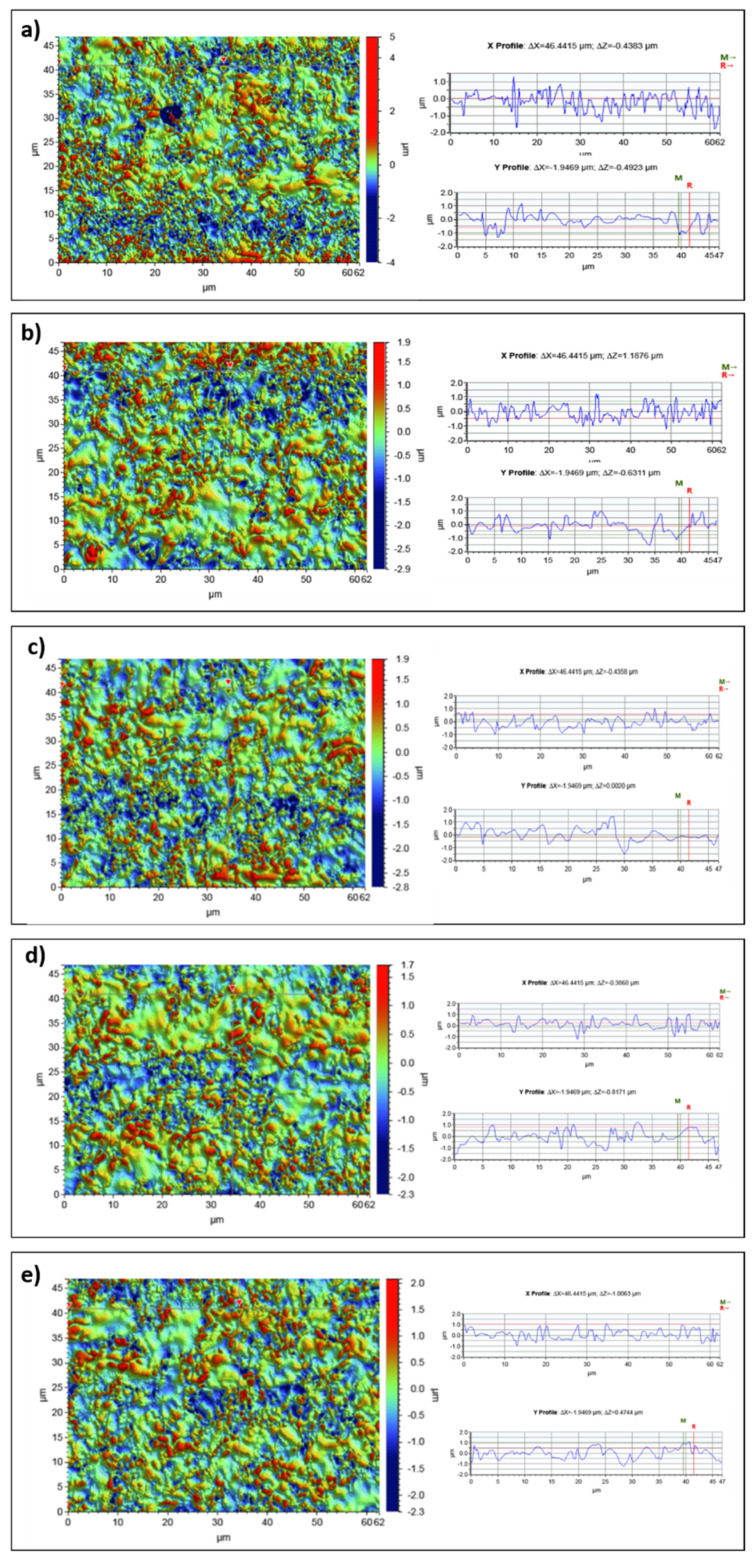
The surface microgeometry maps made for dental implants (**a**) randomly selected site, (**b**) control, (**c**–**e**) irradiation variants, and the height profiles made in the X axis (upper profile) and Y axis (lower profile) for a scan size of 117 µm × 156 µm.

In order to assess the surface roughness of dental implants, tests were performed using optical profilometry before and after laser irradiation. Surface microgeometry maps and linear profiles in the X axis and the Y axis were presented in this study. The following roughness parameters were determined: the arithmetic mean of the elevation profile Ra and the mean square elevation profile Rq and the maximum height of the profile Rt. The research results are presented in Figure 3. 

The determined roughness parameters are presented in the Table 3.

The tests carried out with the use of optical profilometry did not show an increase in the surface roughness parameters of dental implants as a result of laser irradiation. The roughness increase was not observed for any of the irradiation variants. However, a decrease in the roughness parameters Ra, Rq, Rt was observed under the influence of the laser. There were no significant differences in the roughness parameters depending on the exposure variant. The performed linear profiles in the X and Y axes did not show any significant cavities in the tested material. The surface of the tested materials has a uniform roughness over the entire tested area. The tests did not show the destructive effect of laser irradiation on the surfaces of dental implants.

## 4. Discussion

Patients often decide to undergo implantoprosthetic treatment because it allows the missing teeth to be replaced without disturbing the neighboring ones, as was the case with traditional methods of dental prosthetics. Despite the generally large advantage this treatment modality has over other therapies, it also involves a high risk of developing periimplant inflammatory conditions. As the popularity of implant therapy grows, there are more and more patients who experience bone loss around the implants. The causes of periimplantitis may vary, but it is generally believed that the most common one is suboptimal oral hygiene. In many cases, keeping proper oral hygiene is hindered by faulty dental restoration, performed without taking account of the biological spaces of the dental prosthesis, and the insufficient amount of keratinized gingiva tissue. Because implantology is a relatively new field of dentistry, all the factors and possible causes of periimplant inflammation have not yet been fully understood. However, the main identified reason for the development of periimplantitis is believed to be microbial contamination. This is why there is a need for treatments allowing the elimination of microorganisms from the implant and its vicinity, and measures are sought that would exhibit biocidal efficacy without affecting the surrounding tissues.

Dabdoub et al. [6] have shown that the surface of the implant and the implant-abutment interface are the areas that should be taken into consideration as potential pockets of microorganisms when planning decontamination and treatment in periimplantitis. Therefore, our study also analysed microorganisms inhabiting the surface of the crown used as suprastructure and the saliva. The significant correlation between the prevalence of microorganisms in periimplant samples and samples from the inside surface of the suprastructure was also discovered over 20 years ago by Keller et al. [18] In this study, a similar dependence was found in patient 2, while in the other patients only *H. parainfluenzae* appeared on both neighbouring surfaces of the crown and the abutment. Therefore, it is not in every case that a patient has similar microflora on the suprastructure and on the implant.

Schaumann et al. [19] demonstrated that the most abundant microbial groups in supramucosal or supragingival plaques on implants and teeth are the family Streptoccocaceae and the genus *Rothia*, which was also confirmed in the present study.

Periimplantitis is a non-uniform type of infection, involving much complexity and mostly connected with Gram-negative species. Our study, similar to the one carried out by Dabdoub et al. [6] indicated that periimplantitis is associated with several pathogens and opportunistic microorganisms. Opportunistic microorganisms are resistant to antibiotic treatment, and mechanical debridement is not sufficiently effective in the treatment of periimplantitis; for this reason, novel methods of decontamination, such as laser irradiation, give favourable results. Gram-negative bacteria in our study were successfully eliminated in 100% upon three sessions of irradiation.

So far, studies of biocidal efficacy of lasers focused primarily on the assessment of the laser’s biocidal effect against *E. faecalis* in root canals, achieving reduction efficacy of 99.9% [20], 94.94–99.44% [21], 73.96–97.07% [22], and 99.8% [23]. In our study, we used irradiation against *S. pneumoniae* and *S. constellatus*, which belong to the same family of Enterococcaceae, and achieved a reduction of 100% on the abutment and 93.18% to 99.93% on the endosseous fixture.

Implant surfaces can be effectively decontaminated by laser irradiation, but incompetent, uncontrolled use of this method can lead to tissue damage. Laser irradiation causes a rise in temperature which can lead to damage to the bone and ultimately loss of the implant. Monzavi et al. [24] demonstrated that the maximum increase in temperature with the use of Er:YAG is less than 10 °C. Several years later, also Monzavi et al. [25] observed a temperature rise of more than 10 °C when using a diode laser, though the result was not statistically significant. Since in the treatment of periimplantitis laser irradiation of different wavelengths is used, Geminiani et al. [26] performed tests using thermocouples and demonstrated that an 810-nm diode laser with 2 W continuous mode generated a temperature increase of 10 °C after only 14 s, but a 980-nm diode laser produced the same increase in only 12 s. This is why it is extremely important to ensure that the right irradiation dose, mode and duration are selected to achieve a favourable biocidal effect without overheating the target surface. In our study, we used 1-min intervals between subsequent irradiations to prevent the implant surface from overheating.

The nature of the changes in the chemical structure as well as topography and morphology of the surface of studied implants before and after laser irradiation was evaluated by the SEM method. The obtained SEM images showed a fractal, rough structure of the titanium implants which is in agreement with results presented in paper [27]. The studies conducted by Bermingham et al. [28] showed that the Ti 6Al-4V ELI implant alloy contains admixtures of aluminium (6 wt%), vanadium (4 wt%) as well as oxygen and iron. What is more, it was reported that in the microstructure of this type of implant exists lamellar structure of α and β. The structure of the randomly selected site (Figure 2a—magnification 5000×), with the best showing fine acicular α and β forms. The results of the morphology analysis of Ti 6Al-4V ELI presented by Zhang et al. [29] also showed the presence of β-phase existing on the α-matrix. Comparing SEM images obtained by him (made by using backscattered electron micrograph and BSE detector), with images presented in Figure 2, allows us to conclude that white irregular forms sticking to the dark surface are β-phase. The SEM images presented in Figure 2 clearly confirmed the cleaning effect of the laser. What is more, in our studies, using of laser treatment (with all 3 variants) did not destruct the surface of the implants. Additionally, using lasers does not cause an increase in temperature which can change the morphology of α-phase [28].

The surface topography of a dental implant plays an important role in the implantation process [27]. The smooth surface topography of the titanium implant improves contact with the bone and the mechanical properties of the connection [27]. According to the literature [30], the roughness of the implant surface, as well as its chemical composition, has a significant impact on the quantity and quality of dental plaque formation. However, there are no studies showing the dependence of the parameters of the surface roughness of a dental implant on the composition of the resulting biofilm [31,32]. However, in studies devoted to dental implants, surface roughness was identified as an important parameter related to the ability of implant materials to be anchored in bone tissue [33].

In order to assess the surface of dental implants, microscopic examinations were carried out with the use of confocal microscopy, surface microgeometry maps, linear profiles in the X and Y axes and the roughness parameters of the tested materials were determined.

The conducted microscopic examinations with the use of optical microscopy showed changes in fluorescence under the influence of laser light. The tests performed showed an increase in radiation absorption with an increase in the radiation variant of the samples. The microscopic examination also showed a red shift of the fluorescent light. It is most likely the effect of cleaning the tested surface as a result of the action of laser radiation. This is confirmed by microbiological tests and SEM images. Another important conclusion from the conducted research is the information that the applied doses of laser radiation do not destroy dental implants.

Optical profilometry is a very effective and widely used method of examining the surface of various types of materials. It has also found application in processes used in dentistry. This method enables the registration of three-dimensional images of the surface and the determination of metrological parameters. The determined roughness parameters allow for a comprehensive assessment of the surface microgeometry of the tested materials. Surface microgeometries are usually described by the following roughness parameters: the arithmetic mean of the elevation profile Ra, the mean square elevation profile Rq and the maximum height of the profile Rt. The tests carried out with the use of optical profilometry did not show an increase in the surface roughness parameters of dental implants as a result of laser irradiation. However, a decrease in the roughness parameters Ra, Rq, Rt was observed. For the control sample, the determined roughness was Ra = 3.14 µm, after applying 3 × 15 s lasering, the roughness dropped to Ra = 3.07 µm. There were no significant differences in the roughness parameters depending on the exposure variant.

Important information on the impact of laser irradiation was also provided by the determined linear profiles made in the X and Y axes. These profiles also showed no significant losses in the material tested.

The advantage of the method of irradiating dental implants with diode lasers in optimal doses is the fact that it has an effective bactericidal effect, without damaging the decontaminated titanium surfaces.

## Figures and Tables

**Table 1:** Microorganisms identified with MALDI-TOF MS on individual surfaces of the dental implant and crown as well as in the saliva of patients diagnosed with periimplantitis.

	Patient 1			Patient 2			Patient 3		
	Species	NCBI ID	SV	Species	NCBI ID	SV	Species	NCBI ID	SV
Saliva	*Haemophilus parainfluenzae*	729	2.08	*Enterococcus faecalis*	1351	2.06	*Haemophilus* *parainfluenzae*	729	2.19
	*Streptococcus constellatus*	184,250	2.13	*Haemophilus parainfluenzae*	729	2.10	*Rothia aeria*	172,042	2.33
	*Veilonella parvula*	29,466	2.18	*Rothia mucilaginosa*	43,675	2.01	*Streptococcus* *constellatus*	184,250	2.33
				*Streptococcus* *constellatus*	184,250	2.13			
				*Streptococcus oralis*	1303	2.04			
				*Veillonella dispar*	39,778	2.06			
				*Veilonella parvula*	29,466	2.01			
Crown over the implant	*Enterococcus faecalis*	1351	1.95	*Enterococcus faecalis*	1351	2.21	*Actinomyces* *odontolyticus*	1660	2.03
	*Haemophilus parainfluenzae*	729	2.03	*Haemophilus* *parainfluenzae*	729	2.12	*Haemophilus* *parainfluenzae*	729	2.11
	*Rothia mucilaginosa*	43,675	2.27	*Streptococcus oralis*	1303	2.15	*Rothia aeria*	172,042	2.25
	*Streptococcus pneumoniae*	1313	2.11	*Streptococcus* *parasanguinis*	1318	1.79	*Rothia mucilaginosa*	43,675	1.98
	*Streptococcus oralis*	1303	2.17				*Streptococcus oralis*	1303	2.02
							*Streptococcus* *pneumoniae*	1318	2.01
Abutment	*Haemophilus parainfluenzae*	729	2.11	*Candida guilliermondii*	4929	2.12	*Haemophilus* *parainfluenzae*	729	2.13
	*Neisseria subflava*	28,449	2.23	*Enterococcus faecalis*	1351	1.97	*Streptococcus mitis*	28,037	1.90
	*Streptococcus mitis*	28,037	2.31	*Haemophilus* *parainfluenzae*	729	2.01	*Streptococcus oralis*	1303	2.24
	*Veillonella dispar*	39,778	2.18	*Klebsiella pneumoniae*	72,407	2.12	*Streptococcus salivarius*	1304	1.08
	*Veilonella parvula*	29,466	2.34	*Staphylococcus aureus*	46,170	2.16			
				*Streptococcus oralis*	1303	2.36			
				*Veilonella parvula*	29,466	2.51			
Endosseous fixture	*Haemophilus parainfluenzae*	729	2.01	*Enterococcus faecalis*	1351	2.21	*Streptococcus* *constellatus*	184,250	2.33
	*Rothia mucilaginosa*	43,675	1.97	*Haemophilus* *parainfluenzae*	729	2.11			
	*Streptococcus constellatus*	184,250	2.13	*Staphylococcus aureus*	46,170	2.01			
	*Veillonella dispar*	39,778	2.11	*Streptococcus* *constellatus*	184,250	2.24			
	*Streptococcus mitis*	28,037	2.14						
	*Klebsiella oxytoca*	1165	2.01						

SV—score value (level of similarity between the unknown microorganism being examined and the reference species); NCBI ID—sequence record processed by NCBI.

**Table 2:** Percent reduction in the number of microorganisms isolated from the patients with periimplantitis after laser irradiation in 3 variants: L1: 1 × 15 s, L2: 2 × 15 s, L3: 3 × 15 s.

Microorganism			Type of Sample			
		Surface Irradiated	Irradiation Time			
			Unirradiated	L1	L2	L3
			Average Number of Microorganisms (CFU/mL)	Reduction (%)		
Gram-negative bacteria	*Klebsiella oxytoca*	Abutment		86.00 *	100.00 *	100.00 *
		Endosseous fixture	1.6 × 10^6^ ± 5.5 × 10^4^	95.00 *	99.83 *	100.00 *
	*Haemophilus parainfluenze*	Abutment		100.00 *	100.00 *	100.00 *
		Endosseous fixture	1.2 × 10^8^ ± 2.3 × 10^6^	97.48 *	99.20 *	100.00 *
	*Neisseria subflava*	Abutment		100.00 *	100.00 *	100.00 *
		Endosseous fixture	5.4 × 10^3^ ± 1.4 × 10^1^	98.87 *	99.63 *	100.00 *
	*Escherichia coli* ATTC 25922	Abutment		100.00 *	100.00 *	100.00 *
		Endosseous fixture	2.1 × 10^6^ ± 5.7 × 10^4^	99.99 *	100.00 *	100.00 *
Gram-positive bacteria	*Streptococcus pneumoniae*	Abutment		100.00 *	100.00 *	100.00 *
		Endosseous fixture	6.3 × 10^5^ ± 1.7 × 10^4^	93.18 *	94.04 *	99.42 *
	*Rothia mucilaginosa*	Abutment		99.42 *	99.92 *	99.99 *
		Endosseous fixture	1.2 × 10^5^ ± 1.1 × 10^4^	94.23 *	98.39 *	99.65 *
	*Streptococcus constellatus*	Abutment		100.00 *	100.00 *	100.00 *
		Endosseous fixture	6.2 × 10^5^ ± 1.3 × 10^5^	96.15 *	99.15 *	99.93 *
	*Staphylococcus aureus* ATTC 29213	Abutment		100.00 *	100.00 *	100.00 *
		Endosseous fixture	5.8 × 10^8^ ± 8.2 × 10^7^	100.00 *	100.00 *	100.00 *
Fungi	*Candida guilliermondii*	Abutment		71.01 *	99.92 *	99.92 *
		Endosseous fixture	1.3 × 10^4^ ± 2.7 × 10^4^	81.35 *	88.85 *	95.67 *
	*Candida albicans* ATTC 10231	Abutment		100.00 *	100.00 *	100.00 *
		Endosseous fixture	9.1 × 10^4^ ± 6.6 × 10^3^	93.10 *	93.80 *	95.44 *

Mean ± standard deviation; * statistically significant difference versus control sample; ANOVA and LSD at a significance level *p* < 0.05.

**Table 3:** The roughness parameters of dental implants for a scan size of 117 µm × 156 µm.

Area	a	b	c	d	e
Scan Size	117 µm × 156 µm				
Ra(average) (µm)	0.323 ± 0.03	0.314 ± 0.03	0.302 ± 0.03	0.301 ± 0.03	0.307 ± 0.03
Rq(average) (µm)	0.416	0.405	0.391	0.388	0.396
Rt(average) (µm)	9.643	4.401	4.575	4.340	4.166

(a) randomly selected site, (b) control, (c), (d), (e) irradiation variants 1 × 15 s, 2 × 15 s, 3 × 15 s, respectively.

## Data Availability

Not applicable.
